# A New Owl Species of the Genus *Otus* (Aves: Strigidae) from Lombok, Indonesia

**DOI:** 10.1371/journal.pone.0053712

**Published:** 2013-02-13

**Authors:** George Sangster, Ben F. King, Philippe Verbelen, Colin R. Trainor

**Affiliations:** 1 Department of Vertebrate Zoology, Swedish Museum of Natural History, Stockholm, Sweden; 2 Department of Zoology, Stockholm University, Stockholm, Sweden; 3 Ornithology Department, American Museum of Natural History, New York, New York, United States of America; 4 Independent Researcher, Gentbrugge, Belgium; 5 Independent Researcher, Perth, Western Australia, Australia; Monash University, Australia

## Abstract

The avifauna of Indonesia is one of the richest in the world but the taxonomic status of many species remains poorly documented. The sole species of scops owl known from Lombok has long been assigned to the widespread Moluccan Scops Owl *Otus magicus* on the basis of superficial similarities in morphology. Field work in 2003 has shown that the territorial song of the scops owls inhabiting the foothills of Gunung Rinjani differs dramatically from that of *O. magicus* and is more similar to those of Rufescent Scops Owl *O. rufescens* and Singapore Scops Owl *O. cnephaeus*. Detailed comparisons of sound recordings and museum specimens with those of other scops owls in Wallacea and the Indo-Malayan region have confirmed the distinctiveness of the Lombok population. We describe *Otus jolandae* as a new species, the Rinjani Scops Owl. It is locally common at elevations from 25–1350 m. and occurs within Gunung Rinjani National Park. The new species is known from seven specimens collected by Alfred Everett in 1896. *Otus jolandae* represents the first endemic bird species from Lombok.

## Introduction

Owls play an important role in many terrestrial ecosystems [1]. However, despite much study, many aspects of the biology, evolutionary history and taxonomy of owls remain poorly known. The incomplete state of knowledge is underscored by the rapid increase of the number of recognized species of owls, from 146 species in 1975 [2] to 250 species in 2008 [3]. This increase is due both to taxonomic revisions [4]–[6] and new discoveries [7]–[9]. The Old World scops owls *Otus* represent the most speciose genus of owls with 51 currently recognized species [3]. Species limits in scops owls are poorly understood due to their complex pattern of individual and geographic variation, the rarity of many species and subspecies in museum collections, and the lack of detailed and comprehensive taxonomic studies.

In the 1970s, it was discovered that vocalizations of scops owls may offer clues to their taxonomic status and affinities [10]–[12]. Marshall [13] subsequently proposed that species limits in scops owls and other small nightbirds are better inferred from vocalizations than from variation in morphology, and offered a revised classification of the species of *Otus*. In recent years, vocalizations have played an important role in many taxonomic revisions of owls [3], [4], [7], [14], [15]. In the field, vocalizations are often the first clue that a population represents a distinct species [8], [9]. Vocalizations are relevant for species level taxonomic studies of owls for two major reasons. First, as in most other non-passerines, variation in the vocalizations of owls is not learned and thus most likely has a genetic basis. As a consequence, differences in vocalizations may reflect evolutionary history. It has been shown for a number of genera that closely related species which have different vocalizations are also phylogenetically distinct, including *Glaucidium* [14], [16], *Megascops* [15], [17], *Otus* [13], [18] and *Psilopsis* [3], [11], but see [19]. Second, vocalizations of owls are involved in mate choice and species recognition [20]. Therefore, differences in vocalizations may indicate that populations do not recognize each other as potential mates or competitors for mates.

In September 2003, George Sangster and Jolanda A. Luksenburg visited the foothills of Gunung (Mount) Rinjani near Sapit, Lombok, Indonesia to make sound recordings of Large-tailed Nightjars *Caprimulgus macrurus* for a taxonomic study [21]. On 3 September, at dusk they heard the owl-like whistles of several duetting or duelling individuals. Playback and spotlighting allowed them to identify the callers as a scops owl of the *O. magicus/O. manadensis* complex. During the next evening, scops owls with similar vocalizations were heard and seen at a different locality within Gunung Rinjani National Park. Although the scops owls on Lombok have been attributed to Moluccan Scops Owl *O. magicus* by previous authors [22], [23], the songs of the scops owls on Lombok were clearly very different from those of *O. magicus* that Sangster and Luksenburg had heard and recorded only a week before on Flores, and were also noticeably different from those of Sunda Scops Owl *O. lempiji* in Java and Sulawesi Scops Owl *O. m. manadensis* in Sulawesi. Playback of the songs of *O. magicus* and Wallace’s Scops Owl *O. silvicola* (which are both known from the nearby island of Sumbawa) did not result in any acoustic or behavioural response. In contrast, the scops owls responded vigorously to playback of the Lombok songs by singing and approaching the loudspeaker.

Only a few days later, Ben F. King observed and recorded scops owls on Lombok and independently concluded that their vocalizations differ from those of *O. magicus, O. lempiji*, *O. m. manadensis* and other Asian scops owls. Ben King’s observations and recordings were made on 7 September 2003 about 1 km NNW of Sesaot (circa 8°31.5′S, 116°14.5′E) on the SW slope of Gunung Rinjani initially at circa 400 m elevation and subsequently closer to Sesaot at circa 200 m. Both sites are circa 1 km to the west of and outside of the SW boundary of Gunung Rinjani National Park. Fifteen individuals were heard (two of which were observed) during about 4 hours. Playback of the owls calls eventually brought them close enough for a good look. Playback of other owl species calls was not attempted.

**Figure 1:**
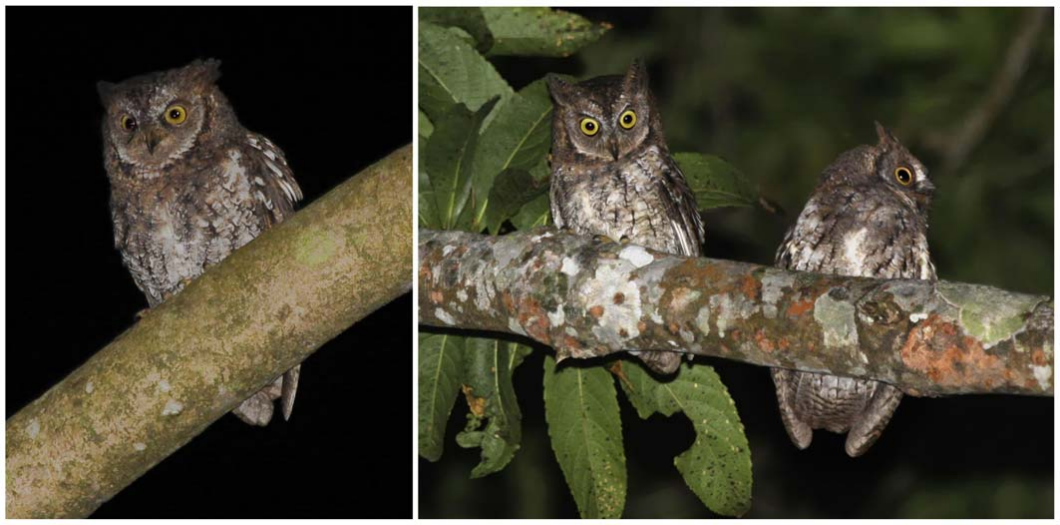
Rinjani Scops Owl *Otus jolandae*, Lombok, August 2008 (Philippe Verbelen).

Aided by sound recordings provided by G.S., Philippe Verbelen and Bram Demeulemeester visited the foothills of the Gunung Rinjani in August 2008 and obtained photographs (Figure 1) and further sound recordings of the owl. They found the species to be common at two localities, Sapit and Senaru. They learned that the owls are known to locals as ‘burung pok’, an onomatopoeic name reflecting the song note of the bird, which may be transcribed as ‘pok’ or ‘poook’.

Finally, Jan van der Laan (in litt.) heard a presumed scops owl on all evenings from 22 to 27 August 2011 in secondary forest at Senggigi in western Lombok at 25 m elevation, and obtained a sound recording.

During our visits to Lombok, no other scops owls were heard. We obtained no evidence for the presence of *O. magicus*, despite playback of the latter’s songs at multiple localities in suitable habitat. Comparison of our photographs of the scops owls on Lombok indicates that these are of the same taxon as a series of specimens obtained on the island by Alfred Everett in 1896 at the same elevations as where we found our scops owl to be common. Detailed comparisons of sound recordings with Indonesian species of *Otus* and comparisons of museum specimens in the American Museum of Natural History, New York (AMNH) and the Natural History Museum, Tring (BMNH) indicate that the Lombok population represents a previously undescribed species.

## Materials and Methods

### Ethics Statement

No specific permits were required for the described field activities or for the visited locations which are not privately-owned or protected in any way. Permissions to visit Gunung Rinjani National Park were obtained locally from park rangers.

### Vocalizations

Recordings from Lombok were included in statistical analyses if visual confirmation was obtained about the identity of the callers (e.g. through playback of recordings). The recorded owls were identified as members of the *Otus magicus/O. manadensis* complex based on a combination of whitish (rather than buff) markings on the belly, lack of a pale nuchal collar and presence of strong dark markings on the underparts (ruling out *O. cnephaeus* and *O. rufescens*, which have vocalizations similar to those of the scops owls on Lombok). Recordings of all known species of *Otus* in Indonesia, Southeast Asia and the Philippines were available for study except *O. alius*, *O. stresemanni*, *O. kalidupae* and *O. siaoensis* for which no recent observations are known [3]. Locations and recordists for all the recordings examined are listed in Table S1. Our recordings from Lombok will be deposited in the Macaulay Library of Natural Sounds, Cornell University.

Characters were defined and assessed on the basis of sonagrams and oscillograms. We recorded measurements of the following 15 variables: F1, frequency at start (Hz); F2, frequency at end (Hz); F3, frequency at 25% of total song duration (Hz); F4, frequency at midpoint (Hz); F5, frequency at 75% of total song duration (Hz); F6, frequency at maximum amplitude (Hz); F7, maximum frequency (Hz); F8, minimum frequency (Hz); DT1, total song duration (s); DT2, time to maximum amplitude (s); DT3, time to maximum frequency (s); DF1, frequency drop from start to end (Hz); DF2, frequency range (Hz); DFT1, slope from 25% to 75% of total song duration (Hz/s); DFT2, slope from midpoint to end (Hz/s). In order to give equal weight to individuals, means of up to five songs were computed for each recording. These means were used as sample points from which ranges, means and standard deviations were computed.

### Morphology

**Figure 2:**
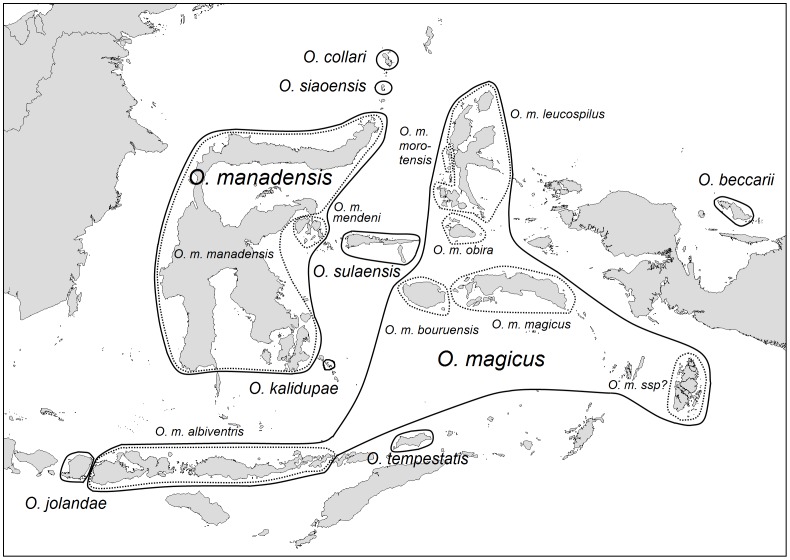
Map of Wallacea and neighbouring islands. Approximate ranges of the species (solid lines) and subspecies (dotted lines) in the *O. magicus* complex are indicated.

To identify morphological differences between the Lombok population and other taxa, we examined 115 specimens of 9 taxa in the *Otus magicus/O. manadensis* complex (Figure 2), and specimens of selected other species of *Otus* occurring in Java and the Lesser Sunda Islands (Table S2). Museum specimens are housed in the American Museum of Natural History, New York (AMNH), the Natural History Museum, Tring (BMNH) and the Swedish Museum of Natural History, Stockholm (NRM). In addition, we examined digital photographs of the unique type of *Otus scops obsti* Eck, 1973.

Whenever possible, we recorded the following information from each specimen: bill length (culmen from skull, from anterior point of nostril), wing length (chord of flattened wing from bend of wing to tip of longest primary), wing formula, shortfall of P4–P10 to tip of longest primary, and tail length (longest rectrix measured from point of insertion of central rectrices to tip of longest rectrix). Mensural characters were measured to the nearest 1 mm. Plumage colours of specimens were compared to published colour standards [24], [25]. Colours in capital letters follow Ridgway [24], whereas those marked with an asterisk follow Smithe [25].

### Statistical Analysis

Principal component analysis (PCA) was used to explore the acoustic and morphometric datasets. PCA reduces multiple variables to a limited number of uncorrelated variables. ANOVA and Tukey’s post-hoc comparisons were used to test whether the groups differed from each other.

Canonical discriminant function analysis (DFA) was applied to the acoustic and morphometric variables to test whether the individuals could be correctly assigned to previously defined groups. DFA generates a set of criteria to assign individuals to groups that are defined prior to the analysis. Prior to DFA analysis, a tolerance test was conducted to assess the independence of each variable. Variables that fail the tolerance test, i.e. which are an almost linear combination of other variables, were excluded from the analyses.

SPSS version 16.0 [26] was used to calculate all descriptive statistics and perform univariate and multivariate analyses.

### Taxonomy

Species and subspecies limits of Old World scops owls are unstable and differ among authorities [3], [23], [27]–[29]. For the purpose of this paper, we follow the most recent classification of König & Weick [3].

### Nomenclatural Acts

The electronic edition of this article conforms to the requirements of the amended International Code of Zoological Nomenclature [30], and hence the new name contained herein is available under that Code from the electronic edition of this article. This published work and the nomenclatural act it contains have been registered in ZooBank, the online registration system for the ICZN. The ZooBank LSIDs (Life Science Identifiers) can be resolved and the associated information viewed through any standard web browser by appending the LSID to the prefix “http://zoobank.org/”. The LSID for this publication is: urn:lsid:zoobank.org:pub:014E23D0-6044-4113-AE53-803838722306. The electronic edition of this work was published in a journal with an ISSN, and has been archived and is available from the following digital repositories: PubMed Central, LOCKSS, ResearchGate, and DIVA (Stockholm University).

## Results

### Vocalizations

**Figure 3:**
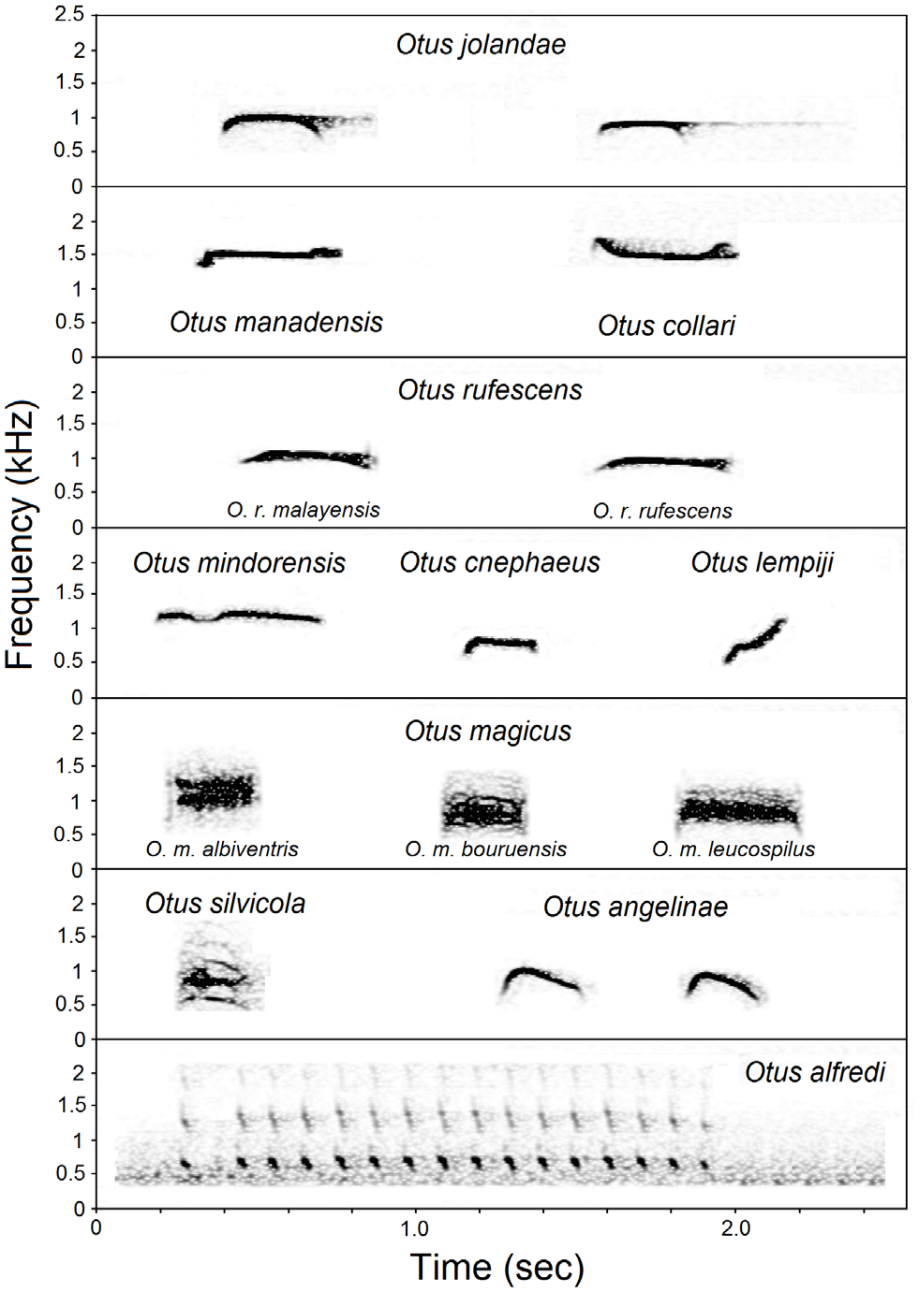
Sonagrams of territorial songs of scops owls. Depicted are songs of *Otus jolandae* (two different individuals), six other species with whistled songs (*O. m. manadensis, O. collari, O. rufescens, O. mindorensis, O. cnephaeus, O. lempiji*), three subspecies of *O. magicus*, and examples of Indonesian owls with songs consisting of a whistle with distinctlive overtones (*O. silvicola*), a double whistle (*O. angelinae*) and a series of multiple notes (*O. alfredi*).

Each currently recognized species of scops owl in Peninsular Malaysia, Indonesia, and the Philippines has a single territorial song type, and these songs may be grouped into five categories: (i) a single whistle without overtones (*O. cnephaeus, O. collari, O. lempiji, O. manadensis manadensis, O. rufescens, O. mindorensis*); (ii) a single or multiple dog- or Raven-like (*Corvus corax*) barks (*O. beccarii*, *O. enganensis*, *O. fuliginosus*, *O. magicus*); (iii) a whistle with distinctive overtones (*O. mantananensis, O. silvicola*); (iv) a double whistle (*O. angelinae, O. longicornis, O. mirus, O. spilocephalus*); (v) a series of multiple notes (*O. alfredi, O. brookii, O. manadensis mendeni*, *O. megalotis, O. mentawi, O. sagittatus, O. sulaensis, O. sunia, O. umbra*). Examples of each of these categories of songs are shown in Figure 3.

Songs from Lombok belong in the first category and consist of a single whistle without overtones, 0.25–0.35 sec in duration, with more or less constant pitch at 1000 Hz (Audio Files S1 and S2). Some song notes show slight frequency modulation. Song characteristics of the Lombok population are given in Table 1 and are illustrated in Figure 3. All recordings from Lombok differed clearly from those of the widespread *O. magicus*, which has a distinctive Raven-like croak (Figure 3, Audio Files S3 and S4). Recordings of *O. m. albiventris* from Flores, *O. m. magicus* from Ambon and Seram, *O. m. bouruensis* from Buru, and *O. m. leucospilus* from Halmahera all have a single note with broadband frequency. The songs recorded on Lombok differ significantly from those of other species of *Otus* with whistled songs in one (*O. rufescens*) to 11 (*O. cnephaeus*) variables (Table 1).

**Table 1:** Characteristics of territorial songs of Indonesian and Malaysian *Otus* with single whistled songs.

Variable^a^	*O. jolandae* (*n = *13)^b^	*O. m. manadensis* (*n = *11)	*O. collari* (*n = *2)	*O. cnephaeus* (*n = *22)	*O. lempiji* (*n = *5)	*O. rufescens* (*n = *7)^c^
F1	864.7±143.5	1179.2±121.9****	1734.8±5.4****	688.3±100.1****	580.4±60.0****	854.2±53.6
	(748.4–1306.0)	(997.4–1350.2)	(1731.0–1738.6)	(547.8–973.7)	(492.8–638.2)	(773.5–938.0)
F2	858.7±128.0	1359.6±151.9****	1701.6±76.9****	730.1±89.6*	997.8±111.8	846.9±67.3
	(720.0–1189.0)	(1122.8–1621.0)	(1647.2–1756.0)	(601.8–904.2)	(853.6–1114.2)	(708.5–916.8)
F3	1029.9±124.7	1306.1±170.0****	1725.1±18.0****	849.9±118.0****	718.7±40.6****	944.6±59.7
	(883.6–1276.0)	(1090.0–1633.0)	(1712.4–1737.8)	(673.6–1121.2)	(671.2–777.2)	(885.7–1052.7)
F4	1018.4±104.0	1312.7±158.6****	1677.9±44.0****	806.7±90.3****	799.8±54.5***	946.0±53.5
	(889.4–1227.0)	(1129.4–1625.5)	(1646.8–1709.0)	(648.2–1007.6)	(748.8–878.4)	(889.8–1047.0)
F5	994.1±122.4	1343.4±127.8****	1653.0±81.2****	783.4±82.1****	925.2±103.5	914.8±50.6
	(826.5–1237.0)	(1163.8–1597.5)	(1595.6–1710.4)	(625.4–915.5)	(814.4–1072.2)	(865.4–1009.7)
F6	992.1±82.2	1333.3±146.0****	1687.8±48.4****	827.4±91.1****	856.9±144.7	945.7±64.9
	(880.8–1172.5)	(1166.0–1624.5)	(1653.5–1722.0)	(699.2–1007.0)	(693.0–1029.4)	(880.8–1046.0)
F7	1056.5±130.2	1417.7±131.2****	1785.7±11.2****	874.4±121.4****	999.5±110.3	961.3±56.0
	(890.6–1322.0)	(1238.2–1678.5)	(1777.8–1793.6)	(720.2–1126.0)	(853.6–1114.2)	(907.6–1071.3)
F8	840.0±121.8	1179.0±125.4****	1642.6±88.2****	678.1±87.3****	580.4±60.0****	827.2±55.5
	(719.2–1189.0)	(997.6–1356.0)	(1580.2–1705.0)	(547.8–895.2)	(492.8–638.2)	(708.5–880.0)
DT1	0.288±0.031	0.340±0.038***	0.511±0.081****	0.218±0.023****	0.169±0.024****	0.412±0.052****
	(0.247–0.343)	(0.261–0.411)	(0.454–0.568)	(0.175–0.273)	(0.143–0.206)	(0.349–0.498)
DT2	0.153±0.049	0.210±0.048	0.203±0.052	0.080±0.034***	0.106±0.054	0.204±0.112
	(0.092–0.228)	(0.144–0.298)	(0.167–0.240)	(0.033–0.140)	(0.038–0.174)	(0.087–0.379)
DT3	0.124±0.059	0.293±0.048****	0.024±0.001	0.065±0.051**	0.169±0.023****	0.145±0.030
	(0.022–0.231)	(0.214–0.379)	(0.024–0.025)	(0.027–0.186)	(0.147–0.206)	(0.118–0.204)
DF1	−6.0±48.7	180.4±78.0****	−33.2±82.3	41.8±76.0	417.4±146.9****	−7.3±55.6
	(−117.0–57.0)	(84.4–296.6)	(−91.4–25.0)	(−144.7–200.8)	(274.0–586.2)	(−65.0–74.6)
DF2	216.5±84.5	238.7±68.5	143.1±77.1	196.2±92.3	419.1±145.0****	134.1±59.2
	(110.0–403.4)	(119.6–343.0)	(88.6–197.6)	(43.7–461.4)	(280.6–586.5)	(78.2–230.0)
DFT1	−244.05±560.02	245.87±453.61****	−265.90±205.16	−591.27±680.02	2482.84±1027.29****	−148.57±112.61
	(−1415.29–364.40)	(−215.45–998.49)	(−410.97 – −120.83)	(−2236.90–805.78)	(1708.49–4254.72)	(−312.65–1.76)
DFT2	−1099.55±486.56	286.55±348.24	103.64±146.55	−713.08±756.97	2344.21±999.24	−498.81±383.49
	(−1877.74 – −274.25)	(−220.10–877.05)	(0.01–207.27)	(−2861.30–330.19)	(1261.25–3713.85)	(−1168.73 – −183.78)

Data presented are mean ± standard deviation (minimum and maximum values). Significance levels of comparisons with *O. jolandae* are indicated with asterisks (* *P*<0.05; ** *P*<0.01; *** *P*<0.005; **** *P*<0.001; ANOVA with Bonferroni correction).

a F1, frequency at start (Hz); F2, frequency at end (Hz); F3, frequency at 25% of total song duration (Hz); F4, frequency at midpoint (Hz); F5, frequency at 75% of total song duration (Hz); F6, frequency at maximum amplitude (Hz); F7, maximum frequency (Hz); F8, minimum frequency (Hz); DT1, total song duration (s); DT2, time to maximum amplitude (s); DT3, time to maximum frequency (s); DF1, frequency drop from start to end (Hz); DF2, frequency range (Hz); DFT1, slope from 25% to 75% of total song duration (Hz/s); DFT2, slope from midpoint to end (Hz/s); ^b^ For F6 and DT2, sample size is 12; ^c^ For F6 and DT2, sample size is 6.

**Figure 4:**
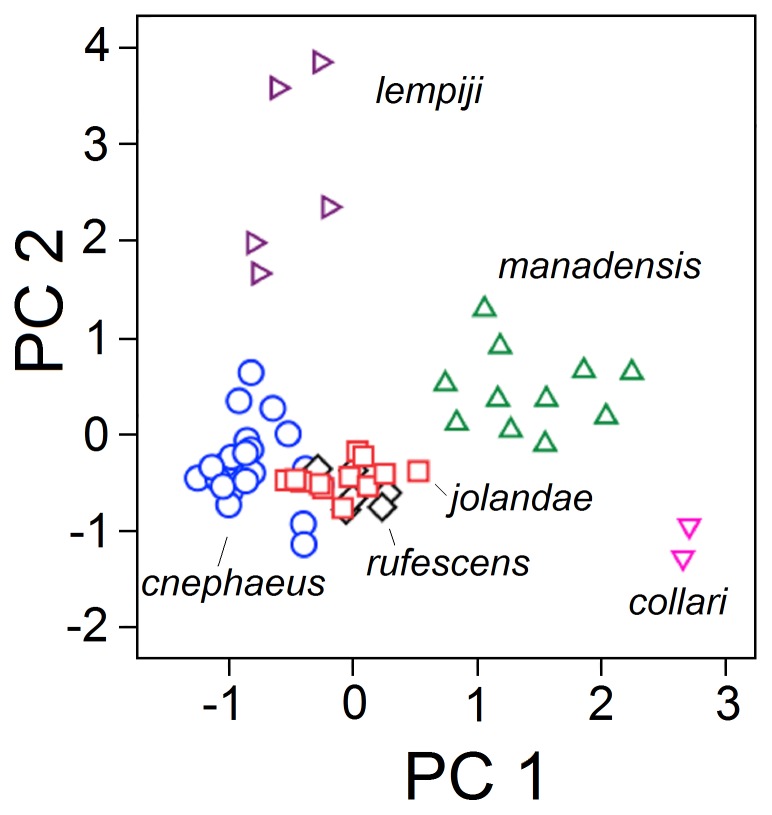
Principal Component Analysis scatterplot of acoustic variables. Depicted are the Eigenvectors of the first and second principal components of 15 acoustic variables measured for territorial songs of *O. jolandae* and five acoustically similar species: *O. rufescens, O. cnephaeus*, *O. lempiji*, *O. collari* and *O. m. manadensis* (*N* = 58).

The songs of 58 individuals of six taxa with whistled songs were used in a PCA (*O. cnephaeus*, *O. collari, O. lempiji*, *O. m. manadensis, O. rufescens*, and the scops owls from Lombok). Three components with eigenvalues >1 were extracted from the data set (Table S3). PC1 was represented by the frequency variables F1–8; PC2 was determined mostly by frequency drop from start to end, frequency range, slope from 25% to 75% of total song duration, and slope from midpoint to end; PC3 mostly by frequency range, time to maximum amplitude, time to maximum frequency, and total song duration. Plotting individuals of all taxa on PC1 versus PC2 revealed four non-overlapping clusters (Figure 4). Three of these correspond to *O. m. manadensis*, *O. collari* and *O. lempiji.* A fourth cluster included all recordings of *O. rufescens, O. cnephaeus*, and the scops owls from Lombok.

The groups differed significantly for all three principal components (one-way ANOVA, Table S3). Each of the six groups differed from the other groups by 1–2 principal components (Tukey’s post-hoc comparisons). All differences remained significant after Bonferroni correction. The recordings from Lombok differed significantly from *O. cnephaeus* in PC1; from *O. lempiji* in PC2; from *O. rufescens* in PC3; from *O. m. manadensis* in PC1 and PC2; and from *O. collari* in PC1.

The songs of 58 individuals were then included in a DFA with the six taxa as group categories. All characters passed the tolerance test, except DF1 (frequency drop from start to end) and DF2 (frequency range), which were excluded from the test. The DFA was highly significant (Wilks’ lambda <0.001; Chi-square_65_ = 392.0; *P*<0.001). The variables most important in the discrimination were: frequency at end, frequency at 25% of song duration, slope from midpoint to end, frequency at 75% of song duration, and maximum frequency (Table S4). The DFA led to a 100% correct classification of the individuals into the six groups. The first of the five discriminant functions accounted for 53.5% of the variation, and the second and third accounted for 26.0% and 10.5% of the variation (Table S4).

### Morphometrics

Morphometric data on the specimens from Lombok and other members of the *O. magicus-O. manadensis* complex are given in Table 2. Morphometrics of the scops owls on Lombok differed significantly from those of *O. m. albiventris* (Sumbawa, Flores, Lembata [formerly Lomblen]), *O. m. magicus*, *O. m. bouruensis*, *O. m. leucospilus* (Moluccas), *O. tempestatis* (Wetar), *O. manadensis manadensis* (Sulawesi) and *O. kalidupae* (Kaledupa I.).

**Table 2:** Measurements of morphological variables for *Otus jolandae* and other members of the *O. magicus-O. manadensis* complex.

Variable	*O. jolandae*	*O. m. albiventris*	*O. m. magicus,* *O. m. bouruensis,* *O. m. leucospilus*	*O. tempestatis*	*O. m. manadensis*	*O. kalidupae*
Bill to skull	21.8±0.7	22.4±1.0	26.3±1.5****	21.8±0.5	21.6±0.7	25.0±1.4
	(7; 21–23)	(8; 21–24)	(21; 24–29)	(8; 21–23)	(27; 20–23)	(2; 24–26)
Bill to nostril	11.9±0.7	10.8±0.7*	13.1±0.8***	10.8±0.7*	10.3±0.4****	11.5±0.7****
	(7; 11–13)	(8; 10–12)	(21; 11–15)	(8; 10–12)	(27; 10–11)	(2; 11–12)
Wing	153.6±3.4	161.3±4.0	175.6±7.0****	151.9±2.9	155.2±4.9	169
	(7; 148–157)	(7; 154–165)	(20; 164–191)	(8; 149–157)	(29; 145–166)	
Tail	74.0±0.9	75.7±4.1	83.3±5.5****	74.3±2.3	72.7±3.5	83
	(6; 73–75)	(6; 70–81)	(20; 74–92)	(8; 70–77)	(29; 67–81)	
Shortfall P10	33.3±2.9	34.0±4.9	41.4±3.7**	38.6±3.7	36.6±3.2	36
	(3; 30–35)	(5; 26–38)	(15; 34–47)	(8; 33–44)	(26; 33–44)	
Shortfall P9	10.4±0.9	11.5±1.8	15.3±1.9****	14.1±1.9*	12.8±2.2	12
	(5; 9–11)	(6; 9–14)	(14; 13–19)	(8; 12–17)	(26; 9–19)	
Shortfall P8	2.2±0.8	4.0±2.8	4.5±1.7*	3.8±1.2	2.5±1.3	1
	(6; 1–3)	(7; 2–10)	(15; 0–7)	(8; 2–6)	(26; 1–6)	
Shortfall P7	0.0±0.0	0.7±1.5	0.2±0.6	0.1±0.4	0.1±0.4	0
	(7; 0–0)	(7; 0–4)	(15; 0–2)	(8; 0–1)	(26; 0–2)	
Shortfall P6	1.7±1.3	0.9±1.2	1.3±1.7	0.6±0.7	2.0±1.1	2
	(7; 0–4)	(7; 0–3)	(15; 0–7)	(8; 0–2)	(23; 0–4)	
Shortfall P5	8.0±4.0	7.0±1.4	6.9±2.9	6.0±1.5	7.4±1.4	9
	(7; 6–17)	(6; 5–9)	(14; 4–16)	(8; 3–8)	(23; 4–10)	
Shortfall P4	14.9±3.4	14.0±2.0	14.1±3.7	13.4±1.4	14.9±1.8	16
	(7; 11–21)	(6; 12–17)	(15; 10–26)	(8; 11–16)	(24; 11–18)	
Wing formulae	6 = 7>8>5>9 (*N* = 1)	6 = 7>8>5>4 (*N* = 1)	6 = 7>8>5>4 = 9 (*N* = 1)	6 = 7>5>8>4 (*N* = 1)	6 = 7>8>5>9 (*N* = 1)	7>8>6>5>9 (*N* = 1)
	7>6 = 8>5>9 (*N* = 2)	6 = 7>8>5>9 (*N* = 1)	6>7>8>5>4 (*N* = 1)	6 = 7>8>5>4 (*N* = 2)	6>7>5>8>4 (*N* = 1)	
	7>6>8>5>9 (*N* = 1)	6>7>5>8>4 (*N* = 1)	7 = 8>6>9>5 (*N* = 1)	6>7>8>5>4 (*N* = 1)	7>6 = 8>5>4 (*N* = 1)	
	7>8>6>5>9 (*N* = 1)	7>6>8>5>9 (*N* = 2)	7>6>5>8>4 (*N* = 1)	7>6 = 8>5>4 = 9 (*N* = 1)	7>6 = 8>5>9 (*N* = 3)	
		7>8>6>5>9 (*N* = 1)	7>6>8>5>4 (*N* = 7)	7>6>8>5>9 (*N* = 3)	7>6>8>5>4 (*N* = 1)	
			7>6>8>5>9 (*N* = 2)		7>6>8>5>4 = 9 (*N* = 2)	
					7>6>8>5>9 (*N* = 5)	
					7>8>6>5>9 (*N* = 9)	

Data presented are mean ± standard deviation (sample size; minimum and maximum values). All measurements are in millimeters. Significance levels of comparisons with *O. jolandae* are indicated with asterisks (* *P*<0.05; ** *P*<0.01; *** *P*<0.005; **** *P*<0.001; ANOVA with Bonferroni correction).

Due to moult, feather abrasion or damage, all but one of the scops owls on Lombok had missing data for at least one variable. Because exclusion of either all variables with missing data or all specimens with missing data precluded meaningful comparisons, an intermediate approach was adopted that maximizes the sample size and number of variables. We excluded two variables (shortfalls of P9 and P10), and one species (*O. kalidupae*, for which only one specimen with complete morphometric data was available) from the dataset. The resulting dataset included 9 variables and 52 specimens, of which five were from Lombok.

In the PCA, three components with eigenvalues >1 were extracted from the data set. Each of the five groups differed significantly (*P*<0.001) from the other groups by the first principal component (one-way ANOVA, with Tukey’s post-hoc comparisons). All differences remained significant after Bonferroni correction. Bill length, wing length and tail length were the most important variables (Table S5).

In the DFA, four discriminant functions were extracted, of which the first explained 84.1% of the variance. All characters passed the tolerance test. The descriptive DFA was highly significant (Wilks’ lambda = 0.026; Chi-square_36_ = 161.4; *P*<0.001). The variables most important in the discrimination were bill length, wing length, and shortfall of P8 (Table S6). Overall, 82.7% of cases were classified correctly to their taxon. Most scops owls on Lombok were correctly classified although one was misclassified as *O. tempestatis*, and one *O. tempestatis* was misclassified as a scops owl from Lombok.

## Discussion

Our results show that the population of scops owls on Lombok differs vocally from all other known species of *Otus*. Its song differs dramatically from those of the different subspecies of *O. magicus*, including that of *O. m. albiventris* to which the Lombok population had been assigned by all previous authors. Ironically, the first clues that the Lombok *Otus* song differs from that of *O. magicus albiventris* were already present in the original report on A. Everett’s specimens. Hartert [31], who reported on Everett’s field work, noted that ”[t]he cry is a clear but not very loud 'pwok,' like that of [*O.*] *lempiji*, but somewhat different in tone”. This is a correct transcription of the song of the owls we recorded on Lombok and is inconsistent with the barking song of *O. magicus*. Photographs of the calling owls (Figure 1) show finely barred belly feathers consistent with specimens collected by A. Everett on Lombok in 1896 and different from those of *O. m. albiventris* (see diagnosis below). Thus, both songs and morphology suggest that our observations and sound recordings of scops owls on Lombok are of the same taxon as that collected by A. Everett.

Interestingly, the song of the scops owls on Lombok is more similar to those of *O. rufescens* and *O. cnephaeus* than to those of any member of the *O. magicus/O. manadensis* complex. Despite the similarities in vocalizations, *O. rufescens* and *O. cnephaeus* are morphologically very different from the scops owls on Lombok, and these three species are likely not closely related.

Taxonomic studies may lead to incorrect conclusions if non-homologous vocalizations are compared. The acoustic repertoire of most owls in the Indo-Malayan region and Wallacea is poorly known. However, our observations strongly suggest that the vocalizations of the Lombok population represent the territorial song. First, the Lombok owls strongly responded to playback of their song by calling back and by flying towards the speaker and displaying above the speaker. These observations are similar to the responses to playback of conspecific songs that we observed in numerous other species in Indonesia and elsewhere (e.g. *O. magicus* on Flores, Buru, Seram and Halmahera, *O. manadensis manadensis* on Sulawesi, *O. manadensis mendeni* on Banggai, and *O. sulaensis* on Taliabu). Second, the vocalizations of the Lombok *Otus* are a pure note which is loud, repetitive, easily located and given from prominent perches, characteristics which are a general feature of territorial songs in owls and nightjars [13], [32].

We conclude that the Lombok scops owls represent a unique lineage, perhaps most closely allied to *O. magicus albiventris* of Sumbawa (only circa 13.5 km east of Lombok), Flores and Lembata, which it closely resembles morphologically. The seven specimens of the Lombok scops owl show dramatically less individual variation than any of the other taxa examined, suggesting a small founder population, perhaps related to a catastrophic eruption of the still active Gunung Rinjani volcano. We found no evidence for the existence of any other species of scops owl on Lombok, nor did Everett’s workers during several months of field work on the island [31]. The type locality of *albiventris* is Flores, and *albiventris* thus remains the valid name for the population of *O. magicus* on Sumbawa, Flores and Lembata.

*Otus scops obsti* Eck, 1973 was described from a single specimen from Java, collected by Wolf Kurt von Schierbrand. The specimen was said to belong to the ‘*manadensis*’ group [33]. However, no population of the *O. magicus/O. manadensis* group is known from Java despite extensive collections made in the 1900s (e.g. by the Bartels family). Based on our examination of photographs of the type of *obsti*, we conclude that this specimen is not a representative of the Lombok population, and differs strongly from the latter in both the pattern and colouration of upper- and underparts (see diagnosis below). Resolution of the true identity of the type of *obsti* probably requires molecular analysis, and is outside the scope of this paper.

The scops owls on Lombok represent a previously undescribed species, which we name:

### *Otus jolandae* sp. nov.

urn:lsid:zoobank.org:act:784483E9-71DB-4071-9B58-A37FA3075116.

#### Holotype (Figure S1)

BMNH 1897.11.1.55. Lombok, 1500 ft, male, collected by A. Everett, May 1896. Label data: iris golden yellow, bill brown, feet dull ochraceous. Previously assigned to *Pisorhina albiventris* [31], *Scops albiventris* (label data), or *Otus magicus albiventris* [34].

#### Paratypes

BMNH 1955.6 N20.3869. N. Lombok, male, collected by A. Everett, June 1896 (Figure S1). BMNH 1897.11.1.56, N. Lombok, female, collected by A. Everett, June 1896. AMNH 629939 Lombok I. 1,500 ft. male, collected by A. Everett, May 1896. AMNH 629940 N. Lombok I. 2,000 ft., male, collected by a native collector for A. Everett, July 1896. AMNH 629941 N. Lombok I., female, collected by a native collector for A. Everett, June 1896. AMNH 629942 N. Lombok I. 2,000 ft., female, collected by a native collector for A. Everett, July 1896.

### Diagnosis: Morphology

*Otus jolandae* is much like *O. m. albiventris*, but differs by: (i) upperparts less boldly patterned with Mummy Brown shaft streaks, short narrow Mummy Brown bars and paler warm brown bars (in *O. m*. *albiventris* the warm brown bars are usually paler, creating a more strongly patterned back, giving a somewhat spotted or barred look); (ii) crown, nape and upperparts dark cinnamon brown (most *O. m. albiventris* have a colder greyer tone); (iii) warm cinnamon breast is darker, with narrow and irregular white and Mummy Brown bars and Mummy Brown shaft streaks (in *O. m. albiventris*, breast feathers usually have more white bars and/or white patches of variable size, resulting in paler breast); (iv) belly feathers usually with more fine dark barring and white patches only on basal half of feathers, except between legs (in *O. m*. *albiventris* belly feathers usually have less fine dark barring and larger white patches, resulting in a paler belly).

*Otus jolandae* differs from *O. tempestatis* by: (i) upperparts more boldly marked with broader dark shaft streaks and bars (in *O. tempestatis*, upperparts are plainer with narrower dark streaks and bars [one red morph female, AMNH 629960, however, has bold blackish shaft streaks which contrast sharply with rufous upperparts]); (ii) crown, nape and upperparts dark cinnamon brown (in *O. tempestatis* either cold grey-brown [grey morph] or dark rufous (red morph]); (iii) dark breast contrasting strongly with whitish belly (*O. tempestatis* has more uniform underparts with much less contrast between breast and belly [in grey morph, breast and belly buffy white to buffy grey, with blackish brown shaft streaks and narrow wavy bars, the shaft streaks narrower on belly; in red morph, breast pale to dark cinnamon with broad blackish shaft streaks, and the belly with narrower black shaft streaks and pale cinnamon and white bars, the cinnamon bars with narrow dark brown edges]).

*Otus jolandae* differs from the Moluccan forms of *Otus magicus* (*O. m. magicus, O. m. bouruensis, O. m. leucospilus*, and *O. m. morotensis*) by: (i) smaller size (Table 2); (ii) tarsometatarsus fully feathered to base of toes (distal part of tarsometatarsus bare in *O. m. magicus* [6–10 mm bare on anterior side of tarsometatarsus], *O. m. bouruensis* [2–8 mm bare], *O. m. leucospilus* [3–16 mm bare], and *O. m. morotensis* [2 mm bare]).

*Otus jolandae* further differs from *O. m. magicus* and *O. m. bouruensis* by: (iii) crown and upperparts less boldly patterned due to darker, warm brown bars and narrower Mummy Brown bars (in *O. m. magicus* and *O. m. bouruensis* dark bars on crown and upperparts are broader, and pale bars are paler, creating a strongly patterned back); (iv) smaller white markings on greater and median upper wing coverts; (v) darker breast, which contrasts more with pale belly (more uniform underparts in *O. m. magicus* and *O. m. bouruensis,* with little or no contrast between breast and belly; (vi) breast and belly feathers usually with narrower Mummy Brown shaft streaks; (vii) breast ground colour warm cinnamon, with narrow white bars and small white spots, as well as Mummy Brown shaft streaks and bars (in *O. m. magicus* [grey morph only] and *O. m. bouruensis* ground colour of breast is much paler [whiter], the feathers with larger white bars and spots, as well as Mummy Brown shaft streaks and bars; red morph *O. m. magicus* underparts pale cinnamon [darker and browner on breast] with broad Mummy Brown shaft streaks and narrow bars, the breast with small white bars and spots, and the belly barred with white).

In addition, *O. jolandae* differs from *O. m. bouruensis* by: (viii) lack of pale collar (*O*. *m. bouruensis* has a band of dark feathers with variable white patches forming a pale collar around upper back, lower hindneck and sides of neck); (ix) darker throat and ear coverts (whiter ground colour with finer dark barring on *O. m. bouruensis*); (x) belly feathers more strongly barred (in *O. m*. *bouruensis*, belly barring sparse, widely spaced and less conspicuous).

*Otus jolandae* also differs from *O. m. leucospilus* by: (iii) dark cinnamon brown upperparts, ranging only to slightly paler cinnamon brown (similar in *O. m. leucospilus*, but ranges to paler cinnamon brown to cold grey-brown, often more strongly patterned with paler spots); (iv) throat darker buffy white to cinnamon buff with dark bars (paler throat buffy white with finer dark bars and/or streaks in *O. m. leucospilus*); (v) warm cinnamon ground colour of breast (in *O. m. leucospilus* ground colour of breast ranges from similar to brighter cinnamon to paler cinnamon to rusty brown to buffy grey); (vi) darker breast (ranges from similar to much paler because of larger white patches on breast feathers on many *O. m. leucospilus*, reducing contrast between breast and paler belly); (vii) paler belly, more clearly demarcated from dark breast (belly colour on *O. m. leucospilus* ranges from similar to darker and contrasting less with breast).

*Otus jolandae* further differs from *O. m. morotensis* by: (iii) outer webs of row of pale scapulars white to pale cinnamon (dark cinnamon, with small white and blackish markings near tips in *O. m. morotensis*); (iv) belly, undertail coverts and legs with white ground colour and contrast between pale belly and dark breast (in *O. m. morotensis* except for pale cinnamon throat, underparts uniformly dark, i.e. entirely dark cinnamon [including undertail coverts and legs], darker and browner on breast, with broad Mummy Brown shaft streaks and narrow short bars, as well as a few pale cinnamon bars on breast, and narrower Mummy Brown shaft streaks and short bars, and pale cinnamon bars on belly and under tail coverts, the tarsi with Mummy Brown markings).

*Otus jolandae* differs from *O. kalidupae* by: (i) smaller size (Table 2); (ii) dark cinnamon brown crown, nape and upperparts (colder grey-brown in *O. kalidupae*); (iii) somewhat broader dark shaft streaks and bars on crown, nape and upperparts; (iv) bold black tips to outer web of pale row of scapulars (tips mottled dark brown and buffy grey in *O. kalidupae*); (v) strong contrast between dark breast and whitish belly (buffy grey breast and belly more uniform in *O. kalidupae*, the belly only somewhat paler than breast); (vi) broader, bolder dark shaft streaks and bars on belly (dark shaft streaks fine and dark bars faint on *O. kalidupae*); (vii) buffy to rusty buff tarsal feathers with indistinct brownish bars/spots (dark buff with distinct narrow dark brown wavy bars in *O. kalidupae*).

Very similar to *O. m. manadensis*, but *O. jolandae* differs from *O. m. manadensis* by: (i) crown, nape and upperparts vary only from dark cinnamon brown to somewhat paler cinnamon brown (range from similar dark cinnamon brown to paler cinnamon brown to colder brownish grey in *O. manadensis*); (ii) warm cinnamon breast, with Mummy Brown shaft streaks and narrow and irregular white and Mummy Brown bars, is often darker and contrasts more with whitish belly (warm cinnamon to buffy grey breast of *O.manadensis* has similar Mummy Brown bars and shaft streaks, but often broader white bars and larger white patches giving the breast a paler appearance, which contrasts less with paler belly); (iii) belly with extensive white patches on basal parts of feathers (less extensive white patches in most *O. manadensis*, resulting in a darker belly which contrasts less with darker breast).

*Otus jolandae* differs from *O. lempiji* and *O. cnephaeus* by: (i) ear tufts dark cinnamon brown with broad blackish shaft streak and narrow blackish bars and some dark rufous on inner vane and base (in *O. lempiji* and *O. cnephaeus* ear tufts buffy white to dark buff, with blackish markings); (ii) crown and nape dark cinnamon brown with narrow to broad blackish shaft streaks and narrow blackish bars (in *O. lempiji* and *O. cnephaeus* crown and nape black to blackish brown [feathers dark buff to brown with blackish shaft streak, bars and markings, and broad black to blackish brown tip] with some buffy brown markings); (iii) lack of buff nuchal collar; (iv) dark cinnamon brown upperparts (colder grey-brown in most *O. lempiji* and *O. cnaephaeus,* usually with some variable black markings on upper back below buff collar); (v) strong contrast between dark breast and pale belly, both with bold dark shaft streaks and narrow dark bars (in *O. lempiji* and *O. cnephaeus*, underparts [breast and belly greyish buff to dull grayish cinnamon to dull dark cinnamon] plainer and more uniform, with little or no contrast between breast and belly, which have blackish shaft streaks, but fine and inconspicuous dark bars); (vi) feathering on tarsometatarsus only reaches base of toes (in *O. lempiji* and *O. cnephaeus* feathering on tarsometatarsus extends over extreme base of toes [1–4 mm]).

*Otus jolandae* differs from *O. rufescens* in: (i) larger size (wing >140 mm in *O. jolandae*; <130 mm in *O. rufescens*); (ii) crown, nape and upperparts dark cinnamon brown with blackish shaft streaks and bars (uniform deep rufescent brown with dark buff arrow-shaped markings, lacking dark streaks and bars in *O. rufescens*,); (iii) bill dark (pale in *O. rufescens*); (iv) facial disc barred white, warm buff and Sepia (plain rufous in *O. rufescens*); (v) breast and belly strongly patterned with dark shaft streaks and bars, the dark breast contrasting strongly with whitish belly (in *O. rufescens* underparts uniform rusty buff to dark cinnamon [somewhat paler on belly and under tail coverts], sparsely spotted dark brown and lacking dark streaks and bars); (vi) dark bars/spots on tarsal feathers (uniform pale buff to pale cinnamon in *O. rufescens*, with only a few faint darker markings).

*Otus jolandae* differs from *O. alfredi* in: (i) patterned dark cinnamon brown crown, nape and upperparts with dark shaft streaks and bars (crown, nape and upperparts plain dark rufous in *O. alfredi*, the feathers of hindneck and upper back with subterminal buffy white marks); (ii) outer vane of pale row of scapulars white to pale cinnamon with blackish tip (white with blackish tip and broad diagonal dark rufous-brown band with blackish border splitting the white patch in *O. alfredi*); (iii) rusty buff to white bars on outer vane of outer primaries (only broader and more widely spaced white notches on *O. alfredi*); (iv) prominent banding on tertials and upper side of tail (banding faint and inconspicuous in *O. alfredi*); (v) longer ear tufts; (vi) bill dark (bill yellow to orange-yellow in *O. alfredi*); (vii) rictal bristles with whitish to buffy bases (rufous bases in *O. alfredi*); (viii) facial disc barred white, warm buff and Sepia (plain rufous in *O. alfredi*); (ix) sides of lower throat and distal ear coverts white with narrow warm buff subterminal band and broad Sepia terminal band, forming white and Sepia border to facial disc (in *O. alfredi* distal ear coverts dark rufous, and feathers of sides of throat white with broad terminal band mixed dark rufous and Sepia); (x) conspicuously patterned underparts with Mummy Brown shaft streaks and bars on breast and belly (plainer, more uniform underparts without dark shaft streaks and bars in *O. alfredi,* the breast dark rufous with some wavy white and narrow brown barring, and the belly paler and duller buffy white with vague irregular pale rusty brown bars and narrow wavy dark brown bars); (xi) dark bars or spots on buffy white to rusty buff legs (no dark bars or spots on buff to cinnamon legs on *O. alfredi*).

*Otus jolandae* differs from *O. silvicola* by: (i) much smaller size (wing <157 mm in *O. jolandae*; 202–251 mm in *O. silvicola,* [3]); (ii) crown, nape and upperparts dark cinnamon brown with narrow dark shaft streaks and bars (in *O. silvicola* upperparts colder greyish brown to brownish grey, with broader dark shaft streaks and bars); (iii) more contrast between darker, more cinnamon breast and whitish belly (in *O. silvicola*, pale greyish cinnamon to pale buffy grey breast only a little darker than sides of belly with white center of belly); (iv) feathering on tarsometatarsus reaches but does not cover base of toes (in *O. silvicola* feathering covers base of toes).

*Otus jolandae* differs from the type of *Otus scops obsti* by: (i) upperparts with warm brown ground colour (cold brown in *obsti*); (ii) crown and upperparts less boldly patterned due to darker, warm brown bars and narrower Mummy Brown bars (in *obsti*, dark bars on crown and upperparts are broader, and pale bars are paler, creating a strongly patterned back); (iii) breast warmer buff, rather clearly demarcated from much whiter belly (not clearly demarcated in *obsti*); (iv) feathers of belly without broad dark brown sidebars or dark brown diamond-shaped markings (present in *obsti*).

### Diagnosis: Vocalizations

The song of *O. jolandae* differs from those of all other species of *Otus* in Indonesia, South East Asia and the Philippines, except *O. m. manadensis*, *O. collari, O. cnephaeus*, *O. lempiji, O. rufescens* and *O. mindorensis,* by consisting of a single whistle without pronounced overtones.

The song of *O. jolandae* differs from those of *O. m. manadensis* and *O. collari* by significantly lower frequency (variables F1–F8, Table 2) and shorter total song duration. The song of *O. jolandae* further differs from that of *O. m. manadensis* by significantly shorter time to maximum frequency, smaller frequency drop from start to end, and by having a (more pronounced) frequency drop from midpoint to end.

The song of *O. jolandae* differs from that of *O. cnephaeus* by significantly higher frequency (variables F1–F8, Table 2), longer total song duration, longer time to maximum amplitude, and longer time to maximum frequency.

The song of *O. jolandae* differs from that of *O. lempiji* by significantly higher frequency (variables F1, F3, F4 and F8, Table 2), longer total song duration, smaller frequency drop from start to end, narrower frequency range, and by lacking a pronounced increase in frequency between 25% to 75% of total song duration and from midpoint to end.

The song of *O. jolandae* differs from that of *O. rufescens* by significantly shorter total song duration.

The song of *O. jolandae* differs from that of *O. mindorensis* in being monosyllabic (*O. mindorensis* has a distinctive di-syllabic song note) (Figure 3).

### Description of the Holotype

Head. Chin and throat feathers white, wavy-barred Mummy Brown. White bars slightly broader than brown bars. Feathers of forehead white, barred Mummy Brown to black. Supercilium to above eye, feathers white with narrow Mummy Brown edges. Crown feathers and ear tufts Tawny-Olive with broad, dark brown (Sepia*) bars and stripe along shaft. Some ear tuft feathers with broad warm buff (between Cinnamon and Tawny-Olive) patches. Nape slightly warmer coloured than crown due to more extensive warm buff (between Cinnamon and Tawny-Olive) patches on nape feathers. Rictal bristles white proximally, blackish distally. Lores and ear coverts wavy-barred white, warm buff and Sepia*. Distal ear coverts, and lower throat feathers white with narrow warm buff subterminal band and broad Sepia* tip, forming white and dark brown border to facial disc. Sides of neck wavy-barred Mummy Brown, buff and white.

#### Upperparts

Feathers of mantle (i.e. upper back), back and rump predominantly warm brown (between Ochraceous-Tawny and Cinnamon-Brown) with Mummy Brown stripe along shaft and fine and indistinct Mummy Brown bars. Uppertail-coverts slightly paler than back and rump, with Tawny-Olive rather than warm brown ground colour. Scapulars as upperparts. A single row of scapulars has Sepia* shaft streak, white outer vane with triangular Sepia* tip bordered by a narrow, warm buff band, and whitish buff inner vane with warm buff tip, narrowly barred with Sepia*.

#### Underparts

Breast feathers warm (rusty) buff with Mummy Brown shaft streak and narrow and irregular white and Mummy Brown bars. Upper belly feathers with Mummy Brown stripe along shaft and white, cinnamon and Mummy Brown bars. Feathers on lower belly and vent with Mummy Brown shaft streak and white and cinnamon patches on proximal part, distal part narrowly barred white and Mummy Brown. Undertail coverts white with Mummy Brown shaft streak and broad cinnamon and narrow Mummy Brown bars. Leg feathered to base of toes. Feathers on tarsometatarsus buff becoming whiter distally, with narrow indistinct Mummy Brown wavy bars. Feathers on tibiotarsus off white, mixed with buff, and with indistinct brownish spots/bars.

#### Wing

Outer vane of outer primaries with broad pale (whitish to dull cinnamon) bars, narrow Mummy Brown bars and broad Tawny-Olive bars. Pale bars become more dull cinnamon (i.e. less white) towards tip. Outer vane of inner primaries similar to those of outer primaries, but pale bars darker, dull cinnamon. Inner vane of primaries dark brown with irregular poorly demarcated Tawny-Olive bars, the innermost ones also with rusty buff notches and patches proximally. Secondaries and tertials dark brown (close to Sayal Brown) vermiculated Mummy Brown with paler, Tawny-Olive bars. Greater, median and lesser upper-wingcoverts cinnamon with dense irregular Mummy Brown barring. Some great upper-wingcoverts with pale, whitish outer edges. Some median coverts with small white markings (1–2 poorly demarcated bars) on outer flag. Visible part of wing lining dark brown with cinnamon buff irregular bars and markings. Tips of longer primary coverts mostly cinnamon buff. Axillaries buffy white.

#### Tail

Upper side of R5 irregularly barred and vermiculated Mummy Brown, Dresden Brown and Tawny-Olive, with bars becoming narrower and more indistinct towards tip. Upper side of R1–R4 with distinct and broad Mummy Brown and Tawny-Olive bars.

### Variation in the Type Series

Male BM 1955.6N20.3869 differs from holotype in: (i) feathers on breast and belly with narrower longitudinal Mummy Brown stripe along shaft; (ii) lower belly from between legs to vent white; (iii) feathers on tarsometatarsus more distinctly barred brown; and (iv) inner vane of P9 dark brown with irregular and poorly demarcated Tawny-Olive bars. Male AMNH 629939 differs from holotype in: buffy white chin and throat with narrow dark wavy bars. Male AMNH 629940 differs from holotype in: (i) cinnamon buff chin and throat with narrow dark wavy bars; and (ii) rictal bristles buff proximally.

Female BMNH 97.11.1.56 differs from from holotype and male BM 1955.6 N20.3869 in: (i) crown feathers and ear tufts with narrower, dark brown (Sepia*) bars and stripe along shaft; (ii) breast feathers paler, and less extensively warm (rusty) buff; and (iii) white markings on some median coverts larger, forming round or oval spots. Female AMNH 629941 differs from holotype in buffy white chin and throat with narrow dark wavy bars. Female AMNH 629942 differs from holotype in: (i) scattered white to buffy white juvenile feathers with Mummy Brown spots and bars on crown and nape; (ii) rictal bristles buff proximally; and (iii) buff chin and throat with narrow dark wavy bars.

### Generic Placement

*Otus jolandae* clearly belongs in *Otus* rather than *Bubo*, *Glaucidium, Athene* or *Ninox* on account of its combination of small size, distinctive ear-tufts, facial disc, short rounded wings, and short tail.

### Distribution

**Figure 5:**
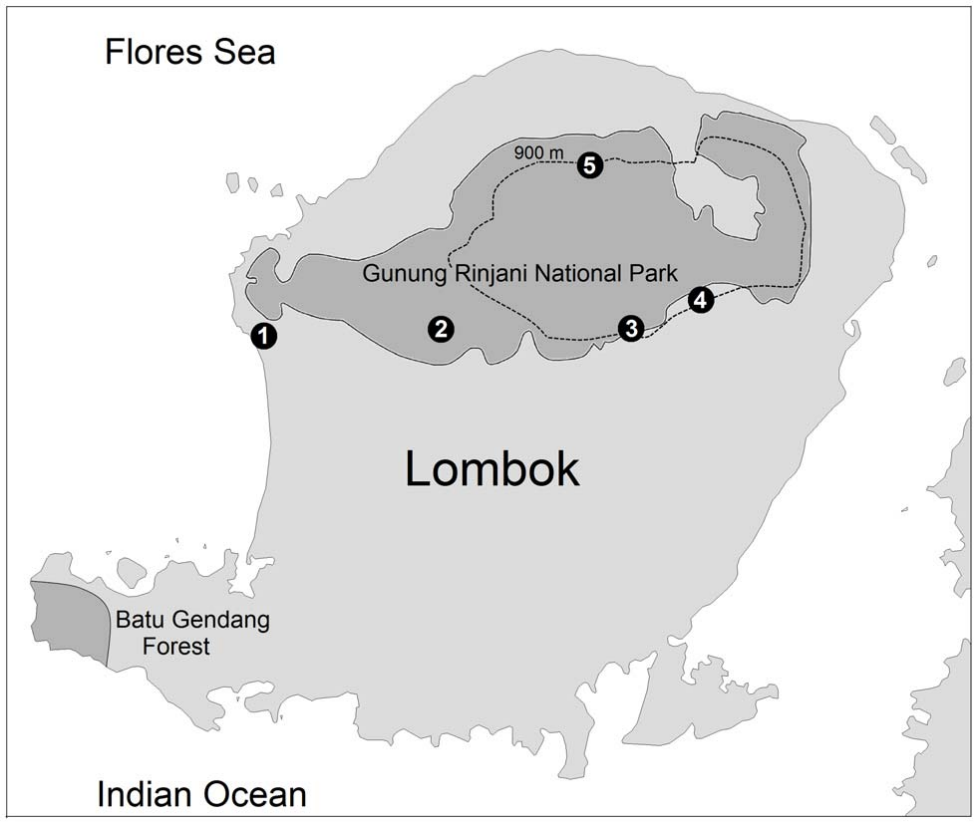
Map of Lombok showing localities where *Otus jolandae* has been recorded. Gunung Rinjani National Park and Batu Gendang Forest are indicated by dark shade. Localities: 1, Senggigi; 2, north-west of Sesaot; 3, Jeruk Manis, north of Kembang Kuning; 4, Sapit; 5, Senaru. The seven specimens collected in 1896 lack precise locality data and are not included in this map.

The seven specimens collected by A. Everett and his personnel lack precise locality data, although label data indicate that some specimens were collected in northern Lombok. Modern records documented by sound recordings are from five localities in western, central and northern Lombok (Figure 5): (i) Senggigi, western Lombok, at 25 m elevation, 22–27 August 2011 (J. van der Laan); (ii) between ½ km NW to 1 km NNW of Sesaot, at 200–400 m elevation on the SW slope of Gunung Rinjani (circa 1 km west and outside the SW boundary of Gunung Rinjani NP), 7 September 2003 (B.F. King); (iii) Jeruk Manis, near the village of Kembang Kuning, at 900 m elevation in Gunung Rinjani NP, 4 September 2003 (G. Sangster, J.A. Luksenburg); (iv) near Sapit, at 900–1000 m elevation, 3 September 2003 (G. Sangster, J.A. Luksenburg) and 27 August 2008 (P. Verbelen, B. Demeulemeester); (v) Senaru, at 500–700 m, and above Senaru along the trail within the forest of the Gunung Rinjani National Park, at 900–1350 m elevation, 28–30 August 2008 (P. Verbelen, B. Demeulemeester). We suspect that *O. jolandae* occurs throughout the forested parts of Lombok, perhaps down to sea level.

Efforts should be made to determine the presence of scops owls in western Sumbawa, where forested habitat can be found at elevations up to 1600 m. The study skins from central Sumbawa (Gunung Tambora) that we have examined all refer to *O. m. albiventris*. *Otus jolandae* was not heard during searches of Gunung Tambora, Sumbawa by Philippe Verbelen in September 2008. Verbelen also conducted a series of interviews with Sumbawan locals in areas comparable to those in Lombok where *O. jolandae* was found. With one exception, none of the locals recognised the songs of *O. jolandae* from playback of recordings made on Lombok. One man recognized the song of *O. jolandae* but he was an immigrant from Lombok who knew the song only from Lombok and had never heard it on Sumbawa.

### Habitat and Biology

**Figure 6:**
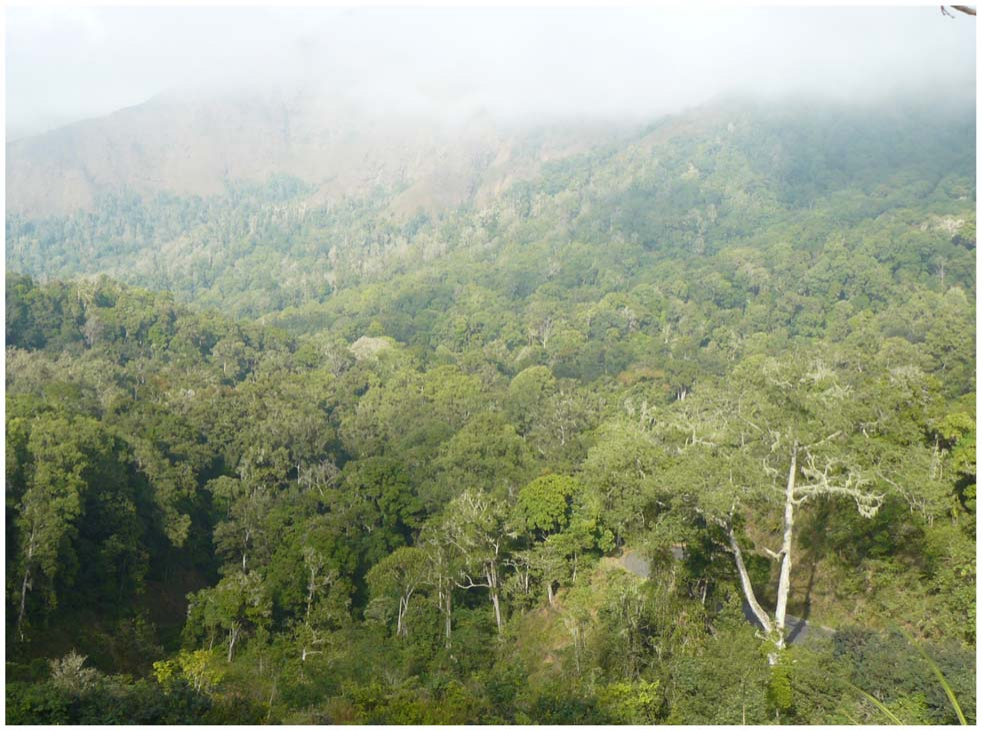
Habitat of *Otus jolandae* in the foothills of Gunung Rinjani, Sapit, Lombok, August 2008 (Philippe Verbelen).

*Otus jolandae* were noted calling from mid-elevation (15–20 m) trees, either within solid forest (Jeruk Manis in Gunung Rinjani NP, north of Sapit in Gunung Rinjani NP, Figure 6) or from patches of trees in more open landscape (Sesaot, Sapit). However, a recording from Senggigi was made in secondary forest with low elevation (7–9 m) palm trees (J. van der Laan in litt.). We recorded *O. jolandae* at elevations from 200–1350 m, but Jan van der Laan’s recording at Senggigi shows that it occurs at least as low as 25 m. Everett’s specimens were collected from “the plains up to about 2000 feet [ = 600 m] above the sea”, although the owls were heard as high up the mountains as 4,500 feet [ = 1350 m] [31]. In September 2003, the owls started calling at dusk between 18∶30 and 18∶40 and continued after dark. In August 2011, one bird was heard calling at 02∶30 (J. van der Laan in litt.). *Otus jolandae* has been heard calling in May-July [31], August (P. Verbelen, B. Demeulemeester) and September (G. Sangster, B. F. King), thus probably territorial during much of the year, as in other tropical owls [35], [36].

### Conservation Status

In the areas where we observed *O. jolandae*, we found it to be common, and up to four individuals could be heard calling from a single spot. We heard *O. jolandae* in undisturbed forest but also in degraded forest, and along quiet roads. Our observations near Sesaot and Sapit with scattered tree patches, and Jan van der Laan’s record in secondary forest at Senggigi, suggest that *O. jolandae* is not dependent on closed primary forest. The species is present in Mount Rinjani NP which covers 413 km^2^ and altitudes ranging from 300–3,726 m. Future studies should determine the exact distribution, elevational range and population density of *O. jolandae* on Lombok, and whether the species occurs throughout the lowlands where extensive forest destruction and cultivation has taken place since the type series was obtained in 1896. Very little lowland forest remains on Lombok, although Batu Gendang forest in Sekotong Tengah (southwest Lombok) still contains stretches of potentially suitable forest.

### Etymology

Named after GS’s wife, Dr Jolanda A. Luksenburg, a biologist at George Mason University, Fairfax, Virginia, USA, who co-discovered the species in 2003 and contributed much to the field work that led to the description of *Caprimulgus meesi* [21].

### Common Name

We propose the English common name ‘Rinjani Scops Owl’ and the Indonesian name ‘Celepuk Rinjani’. Both names refer to Gunung Rinjani, the second highest volcano in Indonesia and Lombok’s most impressive physical feature.

### Previous Records of Scops Owls on Lombok

Very few records of scops owls are known from Lombok, despite several collecting expeditions visiting suitable habitat and elevations. Alfred Russell Wallace did not record any *Otus* on Lombok during his lengthy visits in 1856 [37], nor did Adolphe G. Vorderman in 1894 [38], William Doherty in June 1896 [39], Bernhard Rensch in 1927 [40] and Nagamichi Kuroda in 1929 [41]. Except for the type series collected in May to July 1896 [31], only two observers had recorded scops owls on Lombok prior to our field work in 2003: an individual spotlighted near the losmen at Tetebatu on 14–15 December 1989 [42] and several heard calling (a single-note call) in the northern foothills of Gunung Rinjani, at Pos II (1550 m) along the trail from Senaru to the crater rim, on 14–15 October 1991 (R.W.R.J. Dekker in litt.). Although in both cases no sound recordings were made, it is likely that these referred to *O. jolandae*.

### Biogeography

*Otus jolandae* is the first bird species known to be endemic to Lombok [43], although three endemic subspecies are currently recognised: *Pericrocotus flammeus exul* Wallace, 1864, *Dicaeum maugei neglectum* Hartert, 1897, and *Dicaeum trochileum stresemanni* Rensch, 1928 [22]. In addition, *Trichoglossus haematodus mitchellii* G.R. Gray, 1859 is shared only with Bali, and several other forms are shared only with Sumbawa and Flores [22]. The status of the birds of Lombok has received little attention from taxonomists and the taxonomic validity and rank of the endemic subspecies have not been evaluated recently.

Lombok may have been the only island within the chain of islands from Sumatra to Flores that has remained isolated during glacial maxima in the Pleistocene. At glacial maxima, Bali was contiguous with Java, and Sumbawa was connected through Komodo and Rinca with Flores, leaving Lombok isolated from both [44], [45]. The distribution of scops owls within the chain mirrors this pattern, with *O. lempiji* on Sumatra, Java and Bali, *O. jolandae* on Lombok, and *O. silvicola* and *O. magicus albiventris* on Sumbawa and Flores (*O. m. albiventris* also occurs further east on Lembata). However, current distribution limits may be a poor reflection of historical distribution patterns, and extinction may have played a role in shaping distribution patterns of owls on the Lesser Sundas. For instance, Kusch [46] has found fossil material of *Bubo sumatranus* on Lombok, where the species is not currently present.

## Supporting Information

Figure S1 Photographs of study skins. From left to right: two male *Otus manadensis manadensis* from Sulawesi, two male *O. jolandae* from Lombok (holotype left, male paratype right), and two male *O. magicus albiventris* from southern Flores.
(TIF)

Table S1 Recording localities and recordists. Numbers following each name represent the number of recordings from the recordist at each site.
(DOCX)

Table S2 List of specimens examined.
(DOCX)

Table S3 Factor loadings of 15 acoustic variables on the three principal components in six taxa with whistled songs. Eigenvalues and percentage of variance explained by the respective components are given at the bottom of the table.
(DOCX)

Table S4 Standardized canonical discrimination function coefficients examining trends in variance of 13 acoustic variables^1^ measured for territorial songs of six taxa with whistled songs. Eigenvalues and percentage of variance accounted for by each root are given at the bottom of the table.
(DOCX)

Table S5 Factor loadings of 9 morphometric variables on the three principal components in *O. jolandae*, *O. magicus albiventris*, *O. m. magicus/bouruensis/leucospilus*, *O. tempestatis*, and *O. manadensis manadensis*. Eigenvalues and percentage of variance explained by the respective components are given at the bottom of the table.
(DOCX)

Table S6 Standardized canonical discrimination function coefficients examining trends in variance of 9 morphometric variables measured for *O. jolandae*, *O. magicus albiventris*, *O. m. magicus/bouruensis/leucospilus*, *O. tempestatis*, and *O. manadensis manadensis*. Eigenvalues and percentage of variance accounted for by each root are given at the bottom of the table.
(DOCX)

Audio File S1 *Otus jolandae,* song, Sapit, Lombok, September 2003, George Sangster.
(WAV)

Audio File S2 *Otus jolandae,* song, Senaru, Lombok, August 2008, Philippe Verbelen.
(WAV)

Audio File S3 *Otus magicus albiventris,* song, Riung, Flores, August 2003, George Sangster.
(WAV)

Audio File S4 *Otus magicus albiventris,* song, Labuhanbajo, Flores, August 2003, George Sangster.
(WAV)
