# Supplementary figures and images for: A New Owl Species of the Genus Otus (Aves: Strigidae) from Lombok, Indonesia
Source: PLoS One. 2013 Feb 13;8(2):e53712. doi: 10.1371/journal.pone.0053712 (PMC3572129; doi:10.1371/journal.pone.0053712)

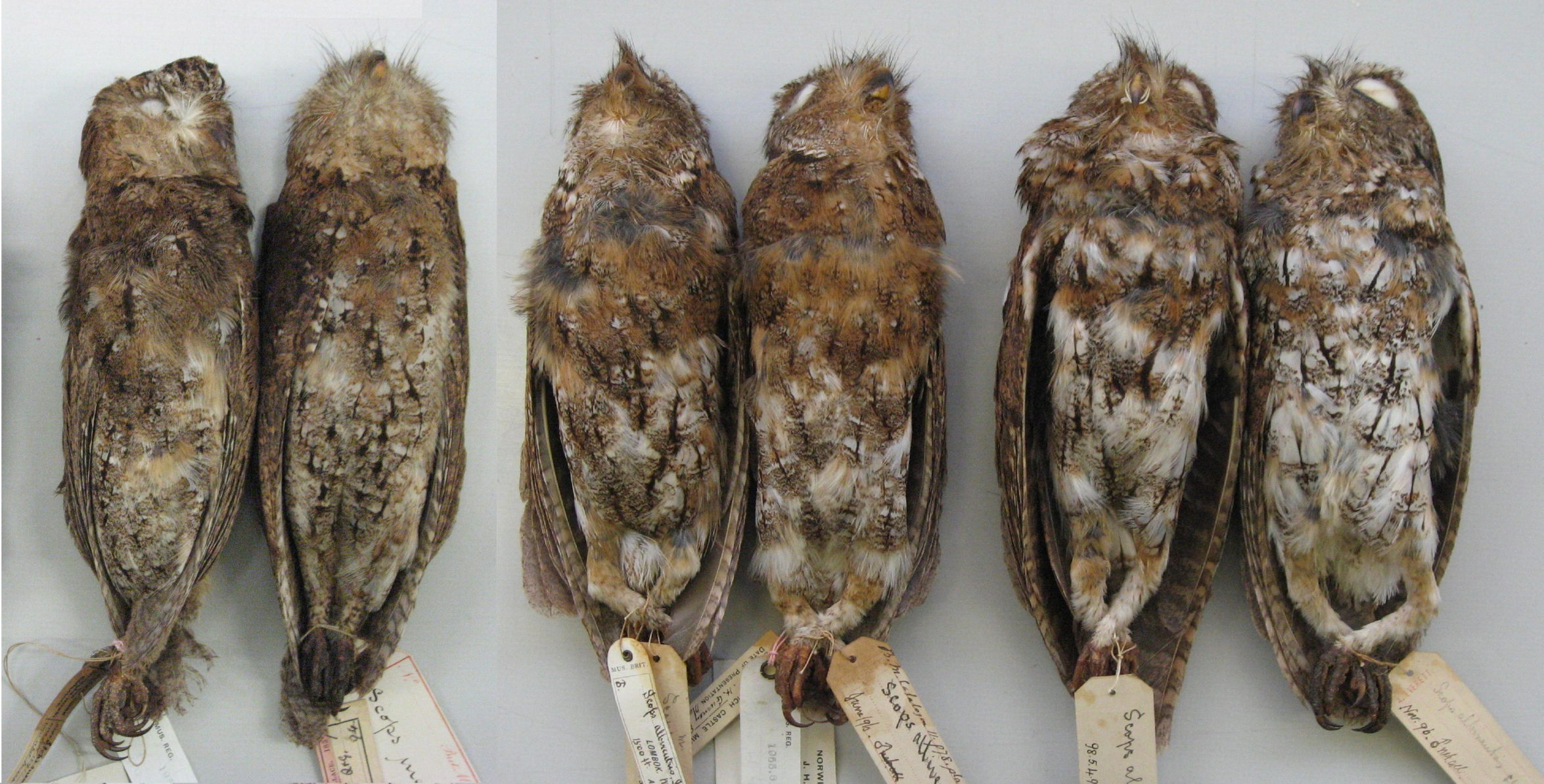

Supplement: Figure S1 — Photographs of study skins. From left to right: two male Otus manadensis manadensis from Sulawesi, two male O. jolandae from Lombok (holotype left, male paratype right), and two male O. magicus albiventris from southern Flores. (TIF) [file pone.0053712.s001.tif]
