# Supplementary material for: A New Owl Species of the Genus Otus (Aves: Strigidae) from Lombok, Indonesia
Source: PLoS One. 2013 Feb 13;8(2):e53712. doi: 10.1371/journal.pone.0053712 (PMC3572129; doi:10.1371/journal.pone.0053712)
Supplement: Table S1 — Recording localities and recordists. Numbers following each name represent the number of recordings from the recordist at each site. (DOCX) [file pone.0053712.s002.docx]

**Table S1.** Recording localities and recordists. Numbers following each name represent the number of recordings from the recordist at each site.

*Otus alfredi* (1 recording). INDONESIA: Danau Ranamese, Flores (B.F. King, 1).

*Otus angelinae* (1 recording). INDONESIA: NNE side of Gng. Gede-Pangrango, Java (J.-H. Becking, 1).

*Otus beccarii* (2 recordings). INDONESIA: Biak (J.T. Marshall, 1; F. Verbelen, 1).

*Otus brookii solokensis* (1 recording). INDONESIA: Gng. Kerinci, Sumatra (S. Lagerveld, 1).

*Otus cnephaeus* (22 recordings). MALAYSIA: Temengor, Belum, Halong MNS base-camp, near Banding, Perak (A.B. van den Berg, 1); Taman Negara, Pahang (S. Buckton, 1; S. Harrap, 1; B.F. King, 1; L. Macaulay, 1; A.B. van den Berg, 1); The Gap, Pahang (G. Sangster, 1); Kuala Selangor, Selangor (N. Gardner, 1; J. Scharringa, 1); Kuala Lumpur (R. Kersley, 4; B.F. King, 2); Tioman Island (G. Sangster, 1). SINGAPORE: Singapore (C. Hails, 2; B.F. King, 1; J.T. Marshall, 1; P. Morris, 2).

*Otus collari* (2 recordings). INDONESIA: Sangihe (F. Lambert, 1; G. Wagner, 1).

*Otus enganensis* (3 recordings). INDONESIA: Enggano I. (B.F. King, 3).

*Otus fuliginosus* (6 recordings). PHILIPPINES: Palawan (D. Fisher, 1; S. Harrap, 1; B.F. King, 1; W. Ruscher Jr, 2; S. Lagerveld, 1).

*Otus jolandae* (6 recordings of 13 individuals). INDONESIA: Sapit, Lombok (G. Sangster, 2, F. Verbelen, 1); Jeruk Manis, near Kembang Kuning, Gng. Rinjani NP, Lombok (G. Sangster, 1); Senaru, Lombok (F. Verbelen, 1); Sesaot, Lombok (B.F. King, 1).

*Otus lempiji* (5 recordings). INDONESIA: Ujung Kulon, Java (B.F. King, 1); Bogor, Java (J.-H. Becking, 1); Mt Salak, Java (J.-H. Becking, 1); Meru Betiri, Java (Bartels & Groeneveld, 1; J.-H. Becking, 1).

*Otus longicornis* (1 recording). PHILIPPINES: Mt Polis, Luzon (J. Scharringa, 1).

*Otus magicus albiventris* (10 recordings). INDONESIA: Maumere, Flores (J.T. Marshall, 1); Riung, Flores (J. Leadley, 2; G. Sangster, 2); Kisol, Flores (A. Lewis, 2); Labuhanbajo, Flores (B.F. King, 1; G. Sangster, 1); Ruteng, Flores (B.F. King, 1).

*Otus magicus bouruensis* (3 recordings). INDONESIA: Wae Hotong, Buru (F. Lambert, 3).

*Otus magicus leucospilus* (13 recordings). INDONESIA: Halmahera (D. Bishop, 1; M. Catsis, 1; R. Drijvers, 1; D. Farrow, 1; N. Gardner, 3; D. Gibbs, 1; A. Greensmith, 1; B.F. King, 1; N. Redman, 1; S. Smith, 1; R. Thomas, 1).

*Otus magicus magicus* (3 recordings). INDONESIA: Ambon (J.T. Marshall, 2); Seram (C.J. Heij, 1).

*Otus manadensis manadensis* (11 recordings). INDONESIA: Tangkoko Batuangus, Sulawesi (M. Catsis, 1; G. Wagner, 1); Dumoga Bone, Sulawesi (R. Bruckert, 1; D. Gibbs, 1); Lore Lindu, Sulawesi (A.B. van den Berg, 2; J. T. Marshall, 1; S. Smith, 1); Rawa Aopa, Sulawesi (A. Skeoch, 1); locality unknown, Sulawesi (G. DeSmet, 1; R. Thomas, 1).

*Otus* *manadensis* *mendeni* (12 recordings). INDONESIA: near Salakan, Peleng (B.F. King, 7); Tolokibit, Banggai (B.F. King, 5).

*Otus mantananensis mantananensis* (1 recording). MALAYSIA: Pulau Mantanani, Sabah (J.T. Marshall, 1).

*Otus mantananensis romblonis* (1 recording). PHILIPPINES: Romblon I. (J.T. Marshall, 1).

*Otus megalotis everetti* (3 recordings). PHILIPPINES: Mindanao (S. Harrap, 1; J. Hornbuckle, 1); Davao, Mindanao (B.F. King, 1)

*Otus megalotis megalotis* (3 recordings). PHILIPPINES: Mt Makiling, Luzon (J.T. Marshall, 1); Mt Polis, Luzon (J. Scharringa, 1); Mt Data, Luzon (G. Wagner, 1).

*Otus mentawi* (7 recordings). INDONESIA: Mentawai Is (B.F. King, 2; J.T. Marshall, 1; F. Verbelen, 4).

*Otus mindorensis* (1 recording). PHILIPPINES: Mt Halcon, Mindoro (B.F. King, 1).

*Otus mirus* (2 recordings). PHILIPPINES: Mt Apo, Mindanao (J. Scharringa, 1; J. Hornbuckle, 1).

*Otus rufescens rufescens* (3 recordings). MALAYSIA: Danum Valley, Sabah (D. Edwards, 1); Poring Hotsprings, Sabah (J. Scharringa, 1); Kinabatangan River (R. Bruckert, 1).

*Otus rufescens malayensis* (4 recordings). MALAYSIA: Taman Negara, Pahang (N. Athanas, 1; J. Scharringa, 1; unknown recordist, 1); Johore (D. Holmes, 1).

*Otus sagittatus* (4 recordings). INDONESIA: Khao Nor Chuchi, Krabi (H. Meijer, 3); Kaeng Krachan NP, Phetchaburi (P.D. Round, 1).

*Otus silvicola* (5 recordings). INDONESIA: Gng. Egon, Flores (R. Drijvers, 1); Rana Mese, Flores (J. Leadley, 2); Ruteng, Flores (J.T. Marshall, 1); Gng. Pacandeki, Flores (S. Smith, 1).

*Otus spilocephalus* (4 recordings). MALAYSIA: Bukit Fraser, Pahang (N. Athanas, 1); Maxwell Hill, Perak (J.T. Marshall, 1); Kinabalu NP, Sabah (J. Scharringa, 1); INDONESIA: Gng. Kerinci, Sumatra (J.T. Marshall, 1).

*Otus sulaensis* (10 recordings). INDONESIA: Taliabu (S. van Balen, 1; P. Davidson, 1; B. F. King, 3; R. Lucking, 2; F. Verbelen, 1); Sanana (D. Yong, 2).

*Otus sunia* (3 recordings). THAILAND: Chiang Mai (J.T. Marshall, 2); Kanchanaburi: Thung Tai (P.D. Round, 1).

*Otus tempestatis* (4 recordings). INDONESIA: Wetar (B.F. King, 2; C.R. Trainor, 2).

*Otus umbra* (3 recordings). INDONESIA: Simeulue I. (J.T. Marshall, 2; M. Lammertink, 1).
