# Supplementary material for: A New Owl Species of the Genus Otus (Aves: Strigidae) from Lombok, Indonesia
Source: PLoS One. 2013 Feb 13;8(2):e53712. doi: 10.1371/journal.pone.0053712 (PMC3572129; doi:10.1371/journal.pone.0053712)
Supplement: Table S2 — List of specimens examined. (DOCX) [file pone.0053712.s003.docx]

**Table S2.** List of specimens examined.

*Otus alfredi* (*N* = 2). INDONESIA: Gng. Repok, S. Flores (AMNH, 1); S. Flores (AMNH, 1).

*Otus cnephaeus* (*N* = 3). MALAYSIA: Teluk Anson, Perak (NRM, 1); Taiping, Perak (NRM, 1); Kuala Lumpur, Selangor (NRM, 1).

*Otus jolandae* (*N* = 7). INDONESIA: Lombok (BMNH, 1; AMNH, 1); N. Lombok (BMNH, 2; AMNH 3).

*Otus kalidupae* (*N* = 2). INDONESIA: Kaledupa I. (AMNH, 2).

*Otus magicus albiventris* (*N* = 10). INDONESIA: Gng. Tambora, Sumbawa (AMNH, 2); S. Flores (AMNH, 3; BMNH, 2); Lomblen (AMNH, 3).

*Otus magicus bouruensis* (*N* = 7). INDONESIA: Mt. Mada, Buru (AMNH, 1); Kajeli, Buru (AMNH, 1); Mt. Madang, Buru (AMNH, 1); Buru? (AMNH, 2); Buru (BMNH, 2).

*Otus magicus magicus* (*N* = 12). INDONESIA: Ambon (AMNH, 1; BMNH, 4); Seram (AMNH, 2; BMNH, 5).

*Otus magicus morotensis* (*N* = 6). INDONESIA: Ternate (BMNH, 2); Morotai (BMNH, 3); Gilolo (BMNH, 1).

*Otus magicus leucospilus* (*N* = 16). INDONESIA: Halmahera (BMNH, 1); Jamkonora, Halmahera (AMNH, 6); S Halmahera (AMNH, 4); N Moluccas? (AMNH, 1); Bacan (AMNH, 3; NRM, 1).

*Otus manadensis manadensis* (*N* = 47). INDONESIA: N. Sulawesi (AMNH, 1; BMNH, 2); Manado, N. Sulawesi (BMNH, 4); Minahasa, N. Sulawesi (AMNH, 2); Mt Masarang, N. Sulawesi (BMNH, 3); Lotta, N. Sulawesi (AMNH, 2); Rurukan, N. Sulawesi (AMNH, 13); Rorokan, Sulawesi (BMNH, 1); Lake Lindu, C. Sulawesi (AMNH, 3); Lombasang, S. Sulawesi (AMNH, 5; BMNH, 1); Indrulaman, S. Sulawesi (AMNH, 1); Talassa Maros, S. Sulawesi (AMNH, 2); Makassar (BMNH, 1); S.E. Sulawesi (AMNH, 1); ‘Sulawesi’ (AMNH, 1; BMNH, 5).

*Otus lempiji* (*N* = 11). INDONESIA: Sukabumi, Gng. Gedeh, Java (AMNH, 2); Bogor, Java (AMNH, 3); Java (BMNH, 5; NRM, 1).

*Otus rufescens rufescens* (*N =* 6). MALAYSIA: Baram, Sarawak (BMNH, 1); Gng. Mulu, Sarawak (AMNH, 1); Benkoker, Sabah (AMNH, 2); Danum Valley, Ulu Segama, Sabah (BMNH, 1); Lairao River, ‘NW Borneo’ (BMNH, 1).

*Otus rufescens malayensis* (*N* = 2). THAILAND: Nakhon Si Thammarat Province (AMNH, 1). MALAYSIA: Gunung Pulai, Johore (BMNH, 1).

*Otus silvicola* (*N* = 3). INDONESIA: S. Flores (AMNH, 3).

*Otus tempestatis* (*N* = 8). INDONESIA: Wetar (AMNH, 8).
