# Supplementary material for: A New Owl Species of the Genus Otus (Aves: Strigidae) from Lombok, Indonesia
Source: PLoS One. 2013 Feb 13;8(2):e53712. doi: 10.1371/journal.pone.0053712 (PMC3572129; doi:10.1371/journal.pone.0053712)
Supplement: Table S3 — Factor loadings of 15 acoustic variables on the three principal components in six taxa with whistled songs. Eigenvalues and percentage of variance explained by the respective components are given at the bottom of the table. (DOCX) [file pone.0053712.s004.docx]

**Table S3.** Factor loadings of 15 acoustic variables on the three principal components in six taxa with whistled songs. Eigenvalues and percentage of variance explained by the respective components are given at the bottom of the table.

| Variable^a^ | PC1 | PC2 | PC3 |
| --- | --- | --- | --- |
| F1 | 0.950 | -0.225 | 0.003 |
| F2 | 0.947 | 0.256 | -0.033 |
| F3 | 0.945 | -0.218 | -0.230 |
| F4 | 0.979 | -0.088 | -0.138 |
| F5 | 0.975 | 0.116 | -0.087 |
| F6 | 0.978 | -0.034 | -0.154 |
| F7 | 0.967 | 0.086 | -0.222 |
| F8 | 0.962 | -0.188 | 0.021 |
| DT1 | 0.736 | -0.377 | 0.339 |
| DT 2 | 0.655 | -0.049 | 0.491 |
| DT 3 | 0.566 | 0.398 | 0.392 |
| DF1 | 0.135 | 0.950 | -0.073 |
| DF2 | 0.046 | 0.650 | -0.579 |
| DFT1 | 0.059 | 0.854 | 0.287 |
| DFT2 | 0.187 | 0.841 | 0.204 |
|  |  |  |  |
| Eigenvalue | 8.773 | 3.295 | 1.129 |
| Variance explained | 58.5% | 22.0% | 7.5% |
| *F* (ANOVA) | 92.627 | 52.954 | 5.315 |
| Significance (ANOVA) | *P* < 0.001 | *P* < 0.001 | *P =* 0.001 |
| Degrees of freedom (ANOVA) | 57 | 57 | 57 |

^a^ F1, frequency at start; F2, frequency at end; F3, frequency at 25% of total song duration; F4, frequency at midpoint; F5, frequency at 75% of total song duration; F6, frequency at maximum amplitude; F7, maximum frequency; F8, minimum frequency; DT1, total song duration; DT2, time to maximum amplitude; DT3, time to maximum frequency; DF1, frequency drop from start to end; DF2, frequency range; DFT1, slope from 25% to 75% of total song duration; DFT2, slope from midpoint to end.
