# Supplementary material for: A New Owl Species of the Genus Otus (Aves: Strigidae) from Lombok, Indonesia
Source: PLoS One. 2013 Feb 13;8(2):e53712. doi: 10.1371/journal.pone.0053712 (PMC3572129; doi:10.1371/journal.pone.0053712)
Supplement: Table S4 — Standardized canonical discrimination function coefficients examining trends in variance of 13 acoustic variables1 measured for territorial songs of six taxa with whistled songs. Eigenvalues and percentage of variance accounted for by each root are given at the bottom of the table. (DOCX) [file pone.0053712.s005.docx]

**Table S4.** Standardized canonical discrimination function coefficients examining trends in variance of 13 acoustic variables^a^ measured for territorial songs of six taxa with whistled songs. Eigenvalues and percentage of variance accounted for by each root are given at the bottom of the table.

| Variable^b^ | Root 1 | Root 2 | Root 3 | Root 4 | Root 5 |
| --- | --- | --- | --- | --- | --- |
| F1 | -0.355 | 0.361 | 0.019 | -0.501 | 0.504 |
| F2 | -2.038 | -0.124 | -0.788 | -4.129 | -2.938 |
| F3 | 1.834 | 0.766 | 0.095 | -2.134 | -0.922 |
| F4 | 0.728 | 0.008 | 2.427 | 3.791 | 3.074 |
| F5 | -0.098 | -0.734 | -2.101 | 1.835 | 0.446 |
| F6 | 0.354 | 0.307 | 0.330 | 0.304 | 0.236 |
| F7 | -0.721 | 0.713 | 0.257 | -0.465 | 0.085 |
| F8 | -0.326 | -0.625 | -0.260 | 0.492 | -0.029 |
| DT1 | -0.811 | -0.583 | 0.550 | 0.185 | -0.590 |
| DT2 | 0.147 | 0.215 | -0.020 | 0.118 | 0.270 |
| DT3 | 0.239 | 0.627 | -0.671 | 0.646 | -0.197 |
| DFT1 | -0.099 | 0.637 | 1.540 | -1.466 | -0.079 |
| DFT2 | 1.633 | 0.673 | 0.682 | 20.634 | 10.367 |
|  |  |  |  |  |  |
| Eigenvalue | 16.514 | 8.030 | 3.226 | 2.365 | 0.705 |
| Variance explained | 53.5% | 26.0% | 10.5% | 7.7% | 2.3% |

^a^ Variables DF1 and DF2 were excluded because these failed the tolerance test.

^b^ F1, frequency at start; F2, frequency at end; F3, frequency at 25% of total song duration; F4, frequency at midpoint; F5, frequency at 75% of total song duration; F6, frequency at maximum amplitude; F7, maximum frequency; F8, minimum frequency; DT1, total song duration; DT2, time to maximum amplitude; DT3, time to maximum frequency; DF1, frequency drop from start to end; DF2, frequency range; DFT1, slope from 25% to 75% of total song duration; DFT2, slope from midpoint to end.
