# Supplementary material for: A New Owl Species of the Genus Otus (Aves: Strigidae) from Lombok, Indonesia
Source: PLoS One. 2013 Feb 13;8(2):e53712. doi: 10.1371/journal.pone.0053712 (PMC3572129; doi:10.1371/journal.pone.0053712)
Supplement: Table S5 — Factor loadings of 9 morphometric variables on the three principal components in O. jolandae, O. magicus albiventris, O. m. magicus/bouruensis/leucospilus, O. tempestatis, and O. manadensis manadensis. Eigenvalues and percentage of variance explained by the respective components are given at the bottom of the table. (DOCX) [file pone.0053712.s006.docx]

**Table S5**. Factor loadings of 9 morphometric variables on the three principal components in *O. jolandae*, *O. magicus albiventris*, *O. m. magicus/bouruensis/leucospilus*, *O. tempestatis*, and *O. manadensis manadensis*. Eigenvalues and percentage of variance explained by the respective components are given at the bottom of the table.

| Variable | PC1 | PC2 | PC3 |
| --- | --- | --- | --- |
| Bill to skull | 0.935 | -0.080 | -0.135 |
| Bill to nostril | 0.893 | 0.028 | -0.219 |
| Wing | 0.934 | 0.030 | 0.037 |
| Tail | 0.908 | 0.016 | -0.056 |
| Shortfall P8 | 0.378 | -0.695 | 0.484 |
| Shortfall P7 | 0.049 | -0.481 | 0.821 |
| Shortfall P6 | 0.035 | 0.857 | 0.122 |
| Shortfall P5 | 0.135 | 0.826 | 0.439 |
| Shortfall P4 | 0.174 | 0.885 | 0.294 |
|  |  |  |  |
| Eigenvalue | 3.565 | 2.922 | 1.274 |
| Variance explained | 39.6% | 32.5% | 14.2% |
| *F* (ANOVA) | 79.646 | 2.008 | 1.067 |
| Significance (ANOVA) | *P* < 0.001 | n.s. | n.s |
| Degrees of freedom (ANOVA) | 51 | 51 | 51 |
