# Supplementary material for: A New Owl Species of the Genus Otus (Aves: Strigidae) from Lombok, Indonesia
Source: PLoS One. 2013 Feb 13;8(2):e53712. doi: 10.1371/journal.pone.0053712 (PMC3572129; doi:10.1371/journal.pone.0053712)
Supplement: Table S6 — Standardized canonical discrimination function coefficients examining trends in variance of 9 morphometric variables measured for O. jolandae, O. magicus albiventris, O. m. magicus/bouruensis/leucospilus, O. tempestatis, and O. manadensis manadensis. Eigenvalues and percentage of variance accounted for by each root are given at the bottom of the table. (DOCX) [file pone.0053712.s007.docx]

**Table S6.** Standardized canonical discrimination function coefficients examining trends in variance of 9 morphometric variables measured for *O. jolandae*, *O. magicus albiventris*, *O. m. magicus/bouruensis/leucospilus*, *O. tempestatis*, and *O. manadensis manadensis*. Eigenvalues and percentage of variance accounted for by each root are given at the bottom of the table.

| Variable | Root 1 | Root 2 | Root 3 | Root 4 |
| --- | --- | --- | --- | --- |
| Bill to skull | 0.517 | -0.520 | 0.093 | 0.557 |
| Bill to nostril | 0.329 | 1.063 | -0.190 | -0.090 |
| Wing | 0.440 | -0.655 | -0.674 | -0.657 |
| Tail | 0.088 | 0.249 | 0.723 | 0.182 |
| Shortfall P8 | 0.460 | 0.112 | 0.801 | -0.077 |
| Shortfall P7 | -0.250 | 0.124 | -0.348 | -0.278 |
| Shortfall P6 | -0.100 | -0.065 | -0.178 | 0.772 |
| Shortfall P5 | 0.231 | -0.283 | -1.381 | -0.602 |
| Shortfall P4 | -0.247 | 0.424 | 1.420 | 0.341 |
|  |  |  |  |  |
| Eigenvalue | 9.441 | 1.111 | 0.396 | 0.272 |
| Variance explained | 84.1% | 9.9% | 3.5% | 2.4% |
